# Regulation of Joint Tissues and Joint Function: Is There Potential for Lessons to Be Learned Regarding Regulatory Control from Joint Hypermobility Syndromes?

**DOI:** 10.3390/ijms26031256

**Published:** 2025-01-31

**Authors:** David A. Hart

**Affiliations:** Department of Surgery, Faculty of Kinesiology and the McCaig Institute for Bone & Joint Research, University of Calgary, Calgary, AB T2N 4N1, Canada; hartd@ucalgary.ca

**Keywords:** joint tissues, joint hypermobility, joints as organ systems, joint tissue regulation, growth and maturation of joints

## Abstract

Normal development of joints starts in utero with the establishment of a cellular and extracellular matrix template. Following birth, individual joint tissues grow and mature in response to biochemical and mechanical signals, leading to a coordinated pattern of further maturation resulting in a joint that functions as an organ system. Each joint develops and matures as an organ system defined by the biomechanical environment in which it will function. For those with joint hypermobility syndromes, either defined by specific genetic mutations or not (i.e., Ehlers–Danlos syndrome, Marfan syndrome, Loey–Dietz syndrome, hypermobility-type Ehlers–Danlos syndrome), this process is partially compromised, but many aspects of joint tissue maturation and resulting joint function is retained such that the organs form and retain partial function, but it is compromised. Comparing the characteristics of what is known regarding development, growth, maturation, and response to stressors such as puberty, pregnancy, and aging in joints of those without and with joint hypermobility leads to the conclusion that in those that have hypermobility syndromes, the joint systems may be compromised via a failure to undergo mechanical maturation, possibly via defective mechanotransduction. Given the breadth of the mutations involved in such hypermobility syndromes, further characterization of this concept may reveal commonalities in their impact on tissue maturation, which will further inform regulatory aspects of normal tissue and functional integrity. This review/perspective piece will attempt to detail such comparisons and summarize how further study will aid in further understanding.

## 1. Purpose of the Review

During fetal development, joints such as the knee, ankle, and shoulder form by cavitation, and the tissues of the joint differentiate and lay down a template of cells and extracellular matrix (ECM) that subsequent growth and maturation will be dependent on to yield a functional joint in the adult. In humans, there can be variation in how a joint such as the knee functions in the context of mobility and navigation. At the macro level, the knee joint, for example, operates as a multi-tissue organ system [1,2,3,4] where each tissue contributes to normal function in an integrated fashion. This whole process is regulated by a combination of biological signals and biomechanical cues, leading to a joint that operates within a physiological “window” around a set point [5]. While many steps in the process leading to a functional joint, for example the knee, in an adult have been investigated over the past several decades, details of how several aspects are regulated still remain to be elucidated.

In contrast to the above scenario, individuals with various forms of joint hypermobility (JH) have joints such as the knee that exhibit excessive laxity or looseness, a condition that can result in pain, increased risk for injury, avoidance of the beneficial benefits of exercise, and increased risk for development of conditions such as osteoarthritis. Many of these individuals have mutations in a variety of ECM molecules, as well as molecules relevant to ECM molecules, while others do not have a defined genotype alteration (reviewed in [6]). These include the multiple forms of Ehlers–Danlos syndrome (EDS) [6,7,8,9], Marfan’s syndrome [10], and Loey–Dietz syndrome (LDS) [11]. In some individuals, some joints are affected more than others for unknown reasons. For many of these individuals, joint hypermobility is just one aspect of altered connective tissue functioning, and other tissues such as skin and elements of the cardiovascular system can be affected to varying degrees, but the latter can have risk for lethal complications such as aortic dissections [12,13,14,15]. The focus of much investigation regarding this population have been on clinical management of pain and rehabilitation [16,17,18] and defining the pathology, as well as the genotyping. However, details of how the various known mutations are modifying joint function, which tissues are affected, and how they are affected at the molecular level remains incomplete.

Given the prevalence of individuals with joint hypermobility (~1 in < 1000) and the breadth of mutations that have been identified (i.e., collagens, proteoglycans, glycoproteins, enzymes, growth factors, and receptors), further investigations providing more detail in JH could also lead to enhanced understanding of how organ systems such as the knee are regulated in the absence of JH during development, growth, and maturation, in response to injury, and during aging and senescence. This perspective/review will attempt to detail some of the information available regarding joints with and without JH, as well as pose questions that could be addressed and how their answers may provide new insights into the regulation of joint tissues that contribute to functioning as an effective organ system across the life span. A focus of the review will be on the knee as an example of a multi-tissue organ that is critical not only for mobility, but also for navigation through the environment.

## 2. Introduction

Development of mobility and the ability to navigate through the environment requires the successful integration of the functioning of a group of tissues that form a joint. Such abilities are essential for the survival of both predator and prey species. Interestingly, species vary greatly in their ability to perform the tasks needed at the time of birth, with some prey species such as zebra (11–13 months gestation) and wildebeest (~8.5 months gestation) able to be up and running within hours of birth, while humans are unable to initiate walking upright until several months of age or even > 1 year in some cases. Why and how such variation is imposed on humans is not well-defined, but it is clear that bipedal mobility and navigation occurs long after the development of joints and are functional at the level of joint motion and crawling on all four limbs. However, the gestation time in the baboon (6 months), chimpanzee (6.5 months), and ape (8–9 months) are similar to humans (~9 months), and like humans, the offspring are born very dependent on their mothers for ~1 year after birth. Therefore, it is the humanoid line not *Homo sapiens* and bipedalism that is unique. Why humans, particularly *Homo sapiens,* are born nearly helpless after 9 months of gestation and can require a year before becoming partially self-reliant regarding mobility and navigation (with straight-line mobility occurring first and then navigation secondly) (discussed in [19]), remains an evolutionary conundrum. However, it is very relevant to understanding subsequent growth and maturation as it pertains to joint functioning. Likely, in part, the timing and sequence of events relate to the integration of joint tissues as an organ system with muscle control via neuro-regulation of the component parts for this species compared to the prey species discussed above.

## 3. Fetal Development of Joint Tissue Organization and Formation of Tissue Templates

Following joint cavitation, the individual joint tissues of an organ such as the knee continue to evolve with specific cells and a rudimentary ECM. This likely happens in the absence of much mechanical loading initially. Thus, the formation of the composite tissue comprised of cells and a template for the subsequent biological and biomechanical growth is mainly dependent on genetics and genetic programming of the associated cells. Thus, at this stage of development, with the organization and composition becoming established, genetic variation due to mutations, splice variants, and variants in promoter regions that may affect rates of expression influence further development. This ECM template likely then serves as a basic form for subsequent addition of matrix molecules during the latter part of gestation and during early growth and maturation following birth. Interestingly, lysyl oxidase, one of the molecules mutated in some forms of JH, is important in collagen assembly and fibril cross-linking in tissues such as tendons [20], and likely others as well as tissues develop.

As joint motion is initiated in utero, with significant growth of the fetus during the third trimester in the human, one has the integration of mechanical loading of joints with the anabolic biologic signals of growth and maturation. This in utero growth prepares the joints for limited function after birth, but the integration of the functioning of the various tissues of a joint, such as the knee, also prepares it to function as a multi-tissue organ system [1,2,3,4].

## 4. Post-Birth Growth and Maturation

After birth, humans are dependent on their mothers for milk and support. While they continue to grow, joints such as the knee are mechanically loaded by motion but not by direct bipedal loading since they do not usually walk upright until 7–13 months of age. Growth of tissues such as ligaments require this motion as immobilization of the knee leads to a failure of the tissue to grow and effectively leads to an arrest of growth at the point of immobilization [21,22]. While it remains to be proven, the immobilization may lead to the cells in such tissues becoming refractory to anabolic growth mediators and factors, possibly by loss of functional receptors for such mediators.

The cells in tissues such as ligaments are arranged in intricate cell networks with the cells connected by connexons [23,24,25]. Early after birth the tissues are cell-rich and ECM-poor, and as they grow, they become ECM-rich and cell poor [26], but the cells form a connected network via the connexins and gap junctions. Thus, prior to birth the template for the ECM and the cell network form for each tissue of the knee, with the added input of the small amount of essential innervation in preparation for the post-birth growth and maturation.

In other tissues such as synovium, the tissue contains a number of collagen types that can form fibrils of different sizes [27]. These collagens include types I, III, V, and VI. As the synovium serves functions other than primarily load dissipation or joint stability, the organization and composition are different from ligaments and tendons. The synovium of the knee is also rich in glycosaminoglycans in addition to collagens [28].

Joint capsules are complex tissues that are highly innervated (discussed in [29]). The tissues are complex with regard to composition and are dynamic in that they change with age [29], and some aspects of the tissues are joint specific [30].

The tissue of an organ system such as the knee appears to grow in a coordinated manner in response to growth of bone as the driver [22,31]. Thus, the multitude of tissues in the functioning knee (i.e., ligaments, menisci, articular cartilage, synovium, fat pads, capsule, muscles, tendons) must respond to the stresses imposed by the growing tibia and femur with coordinated growth to retain function. This is accomplished by the responses of endogenous cells in each tissue to mechanical loading, anabolic mediators, and neural input (all tissues are innervated except the articular cartilage and parts of the menisci). The integration of the responses leads to a strengthening of the various tissues using the templates laid down during development.

**Figure 1:**
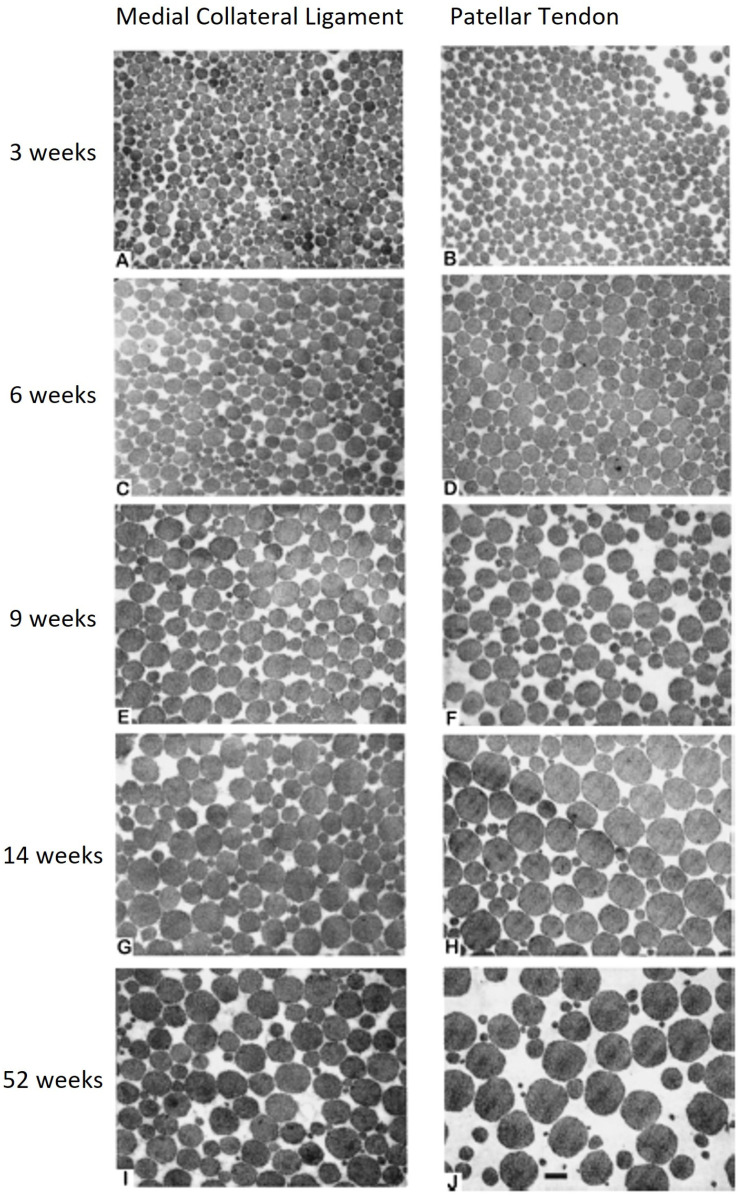
Maturation of the matrix of ligament and tendon with age. Collagen fibril diameters in the rabbit medial collateral ligament (**A**,**C**,**E**,**G**,**I**) and patellar tendon (**B**,**D**,**F**,**H**,**J**) with increasing age from 3, 6, 9, 14 and 52 weeks (top to bottom, respectively). Reprinted with permission from Lo et al. [32].

During this period of growth, mechanically active soft tissues such as ligaments and tendons of the knee not only grow stronger, building on the template formed previously, but also structurally adapt to increasing loading demands by altering the size of their collagen fibrils [32]. This is shown in Figure 1 for the rabbit MCL and patellar tendon (PT), and it has also been shown for other tissues of various joints in a variety of species. Interestingly, as the tissues grow and meet the increasing demand of the mechanical environment, one sees a shift from a unimodal distribution of small-diameter collagen fibrils to a bimodal distribution of large- and small-diameter collagen fibrils. Furthermore, as the MCL is a stabilizing ligament of the knee and one that operates in a lower load environment than does the PT, which operates in a high load environment, the large collagen fibrils in the PT are somewhat larger than those in the MCL, likely indicating that both the size and distribution of fibril diameters is tissue dependent, and one cannot extrapolate or generalize from what is found in one tissue. Therefore, ligaments and tendons may appear to be similar in some regards, they are regulated differently regarding the structure of the ECM [33]. Thus, while building on a template for each tissue laid down during development, tissues of the knee and others have the flexibility to adapt to the mechanical environment. This likely also relates to gene expression patterns for specific molecules (i.e., collagen subtypes, small and large proteoglycans, enzymes affecting collagen cross-linking such as lysyl oxidases, and others). For example, lysyl oxidase is required for collagen fibrillogeneisis by tendon cells [34].

There are a large number of collagens [35], with some of them readily forming fibrils and others serving unique roles in the musculoskeletal (MSK) system. Some types of collagens can bind to fibrils, as do proteoglycans [36,37,38]. When assembled, fibrillar collagens are long molecules with crimp in their structure [39]. This crimp in the collagen serves a purpose as when a tissue is loaded [40,41], the initial response is to straighten the molecules with the crimp taking up the initial load. In ligaments and tendons, the organization is hierarchical with assembly of the collagen into fibrils of varying diameters. When loaded, the fibrils and fascicles appear to be able to slide, facilitated by the boundary lubricant PRG4 around them [42,43,44,45], and expression of PRG4 appears to be affected by age [46] and surgical menopause [47].

How the transition from small collagen fibrils to large fibrils and with a bimodal distribution is formed and regulated into adulthood is not well understood. Interestingly, in knockout mice for small leucine-rich proteoglycans (SLRPs) one can get alterations collagen fibril diameters with lumican, biglycan, decorin, or fibromodulin knockouts [48,49,50,51]. In an analogous set of studies using decorin anti-sense approaches [52], collagen fibril diameters in healing rabbit MCLs were greater in the treated tissues than in those treated with the control antisense reagents [53]. As these SLRP bind to collagen type I in the molecular groove of the assembled collagen molecule [54,55], they may affect the assembly of collagen via this route, but it remains to be determined how such a process is regulated by mechanical loading. In addition, it is challenging to assign “cause and effect” using this approach due to the complexity of the in vivo environment [56]. “Naturally” occurring variation in proteoglycans, including the SLRPs in cranial cruciate ligaments of different dog breeds, has been associated with risk for knee injury [57], but whether the differences translated into mechanical characteristics was not reported.

During this period of growth and maturation from birth to just prior to the onset of puberty, the set point or boundary conditions for a given joint is likely somewhat flexible to accommodate the rate of growth and maturation. There is also detectable variation in the alignment of components of the motion segments comprising the ankle-knee and hip (i.e., for the knee; genu varum, genu valgum). Thus, there is also normal variation in how a motion segment such as a leg functions, a finding that implies that such variation can still lead to a functional knee that can operate successfully across the life span.

As a general rule, joints of young individuals in this age bracket are usually more lax or loose than those of an adult [58,59]. This may be teleologically advantageous to allow for flexibility in maintaining functionality and coordinated growth of a joint during a period of rapid growth with increasing mechanical demands. However, it is not well-defined as to which tissues are the major contributors to joint laxity. Based on function, one could likely conclude for the knee that ligaments such as the ACL, PCL, and MCL, as well as the joint capsule, are involved in the regulation of joint laxity.

Also, in this age bracket many characteristics of joints do not exhibit sex differences. Both sexes exhibit lax or loose joints, and functionally, they are very similar. For example, prior to the onset of puberty, when boys and girls jump off of a box or a height, they land very similarly [60]. Teleologically, this similarity in somewhat lax joints and in functionality likely makes sense as the tissues are growing larger and stronger during this pre-puberty time frame for both boys and girls. Having flexibility in the joints may allow for some variation in the growth rate of different tissues and retain some boundary conditions for the tissues to function as an organ system.

## 5. Onset of Puberty

With the onset of puberty, many changes in connective tissues of the joints start to occur. As all of these tissues contain sex hormone receptors [61,62], they can and do become responsive to the inset of elevated expression of sex hormones such as estrogen, progesterone, and testosterone. Accompanying the onset of puberty is growth in both sexes, with males also becoming more muscular, a process that can affect the functioning of joints. For females, the onset of puberty results in the development of menstrual cycles with fluctuating hormone levels occurring every ~30 days. As mentioned above, before puberty, girls and boys land after a jump with very similar characteristics. However, after puberty, the females change their landing from a jump [59], likely as a result of possible widening of the hips and other structural changes that can occur to facilitate reproductive activities. Therefore, the onset of puberty can have a significant impact on the structure and function of joints, particularly for females. During the menstrual cycle, joint laxity can vary [63,64], corresponding with changes in sex hormone levels. However, not all females experience such changes, and a subset of females do not undergo alterations to joint laxity even though they express changes in sex hormone levels similar to those that do experience alterations in joint laxity [63,64]. Why and how this susceptibility or resistance to changing levels of sex hormones is manifested remains unknown but should be the focus of future investigation. However, there are no known consequences associated with this dichotomy, but this has not been investigated to the author’s knowledge.

With the onset of puberty for females, there is also higher risk for development of joint injuries and abnormal joint maturation. For the latter, an example is the development of Adolescent Idiopathic Scoliosis (AID), where the intervertebral column and associated discs develop abnormal curvatures, which can lead to pathology and disability. Approximately 80–90% of such patients are female [65,66,67]. The cause of this condition is not well-known, but abnormal muscle loading of intervertebral discs could be one explanation, as well as genetic variables and hormone effects as it usually arises after puberty.

There are also reports that adolescent females, and females in general, are at higher risk for knee injuries than their male counterparts [68,69,70]. In particular, the incidence of ACL tears is much higher in females than males during participation in sports that require cutting maneuvers such as soccer and basketball [71]. In part, this has been attributed to variation in knee laxity during the menstrual cycle (reviewed in [70]) and/or due to an imbalance between the quadricep and hamstring muscles [70,71,72,73]. The latter can be corrected (i.e., lowering rates to levels observed for males in the same sports] using neuromuscular training programs such as the FIFA-11+ program [74,75], but it requires continuous participation in such programs, or the muscle imbalance reverts to pre-exercise relationships and an increase in injury risk again. Why this muscle imbalance arises and how it is maintained is not known in detail, but likely it does involve neuroregulatory abnormalities to account for its reversibility after stopping the exercise programs.

A second possibility for the increased risk for knee injuries relates to changes that could result in a decline in efficacy of regulatory mechanisms controlling the integrated function, leading to increased risk for injuries such as ACL tearing (discussed in [63,64,76]). However, knee ligaments and the capsule are likely central to the changes based on their function. Relevant to this point are reports that estrogen can inhibit lysyl oxidase in engineered ligaments and decreases the mechanical function of the tissues in a dose-dependent manner [77]. However, some doses used in that study were in excess of physiological levels. In addition, the authors did not assess collagen fibril diameters in their study. These observations are of interest as lysyl oxidase is a regulator of collagen fibril diameters [34] and thus, the mechanical properties of the tissue.

Serum relaxin levels have also been implicated in ACL injuries (reviewed in [76] and hip injuries [78]). Relaxin levels are reported to vary across the menstrual cycle with the highest levels corresponding to a peak associated with highest risk for ACL injuries. While the ACL does have relaxin receptors (discussed in [76]), and thus, is potentially responsive to relaxin levels changes, presently this is mainly a correlation in humans. However, relaxin and its receptors have also been implicated in cranial cruciate ligament disease in dogs as well [79], so the findings in humans should be followed up with further investigations. As relaxin can regulate matrix metalloproteinase-1, which can cleave collagen I (discussed in [76,78]), the major collagen of ligaments and tendons, it could play a role regulating the integrity of these tissues.

Based on the above discussion, the onset of puberty can lead to many changes in joint tissues and joint function, predominately in females where changes in knee joint laxity or looseness have been well-documented. However, the heterogeneity in females in many aspects has not been well-explained, and several aspects regarding regulation remain to be elucidated by further research. Central to addressing the unknown aspects of joint laxity or mobility is identification of which tissues are involved. While the ligaments of the knee and other joints have been implicated based on their function, whether this also involves the joint capsule, which is a highly innervated tissue [29,80], or others such as muscles remains to be determined.

## 6. Following Skeletal Maturity

Skeletal maturity, defined as the cessation of growth, leads to closing of growth plates in bone and the establishment of a “set point” for how tissues in a joint will interact and function during mobility activities and navigation through the environment [19]. This set point for each joint will be established in an individualized manner based on genetics, epigenetics, history of injury, and activity levels, and includes joint laxity as one integrated functional characteristic.

In contrast to males, females will have sex-dependent influences on joint functioning and joint laxity. These are related to the menstrual cycle previously discussed, and pregnancy. During a 9-month pregnancy, the female has an increasing weight of the fetus to contend with, as well as a number of metabolic alterations to the maternal system. These changes can lead to increases in ligament [81,82] and joint laxity [83], mainly in the third trimester but also occurring earlier. Some reports have implicated the cytokine relaxin in these changes to joint laxity, but this has been challenging to confirm (discussed in [83,84]). Initially, it was thought that the primary function of relaxin was to induce laxity of joints involved in delivery of the fetus. However, reports with knockout mice have indicated that delivery of pups is not impeded in such mice, but aspects of lactation may be compromised [85]. However, this is controversial [86]. Both the Zhao et al. [85] and Parry et al. [86] studies used knockout mice involving the C57BL/6 strain of mice, but the mice used by Zhao et al. [85] used mice with a C57BL/10ScSn genetic contribution, but it remains to be determined whether this difference could account for the differences in outcome. Further to a role for relaxin in pregnancy, Schauberger et al. [83] reported that the changes in joint laxity during pregnancy did not correlate with relaxin levels. However, relaxin levels did correlate with risk for knee [69,76] and hip injury [78]. Thus, further investigation of whether these are clinical important correlations or just associations is required.

In an attempt to better understand the impact of pregnancy on cells and tissues of joints, a rabbit model and an assessment of mRNA levels for a large panel of molecules relevant to each tissue in the knee was used. The tissues investigated include the ligaments of the knee during a first pregnancy in adolescent rabbits [87] and older multiparous rabbits [88], articular cartilage [89], menisci [90], synovium [91], and intervertebral discs (Hart, unpublished observations). For some of the changes observed, there were differences between primigravida and multiparous animals, indicating that changes occurring after the initial pregnancy perhaps did not return to pre-pregnancy conditions. Of significance, mRNA levels for collagens, including collagens I and III, were depressed in pregnant animals, as was a chaperone for collagens HSP47 [92]. This chaperone for collagens serves an important function with regard to collagen assembly [93], as well as the secretion of SLRPs such as decorin, lumican and fibromodulin [94]. Based on such studies, it was clear that pregnancy led to significant modifications to the metabolic activity of cells in tissues of the knee. However, what was not done was to assess whether these metabolic changes led to altered tissue function such as their biomechanical characteristics and whether they correlated with specific alterations in some ECM molecules. Previously, it was shown that the low load behavior of the MCL was increased during pregnancy, but the high load behavior was not altered (reviewed in [5]). As the low load behavior is related to laxity and proteoglycans, while the high load behavior is more aligned with the collagen fibril structure of normal ligament [95], some of the changes in expression of proteoglycans during pregnancy, such as decorin, biglycan, and lumican, may be relevant to collagen structure in such tissues. However, one may have to be somewhat conservative in attempting to correlate molecular changes in cells of joint tissues during pregnancy with biomechanical changes in the tissues. This point was emphasized in studies by Hart et al. [96], where it was demonstrated that many cellular changes were noted during pregnancy in the injured MCL of the rabbit, but there were no detectable changes in the biomechanical properties of the MCL. Thus, there was a disconnect between molecular alterations during pregnancy, and their impact on the function of the tissue!

Thus, the cells in joint tissues are affected at the level of the transcriptome during pregnancy, but the relationship of those changes to altered function remains to be elucidated. In addition, what is contributing to the cellular changes is also not well-characterized. The changes could be mediated by cytokines and hormones such as estrogen or others altered during pregnancy. Alternatively, some of the changes could be mediated by alterations to neuroregulatory elements. As all tissues of the knee and other joints are innervated except for articular cartilage and parts of the menisci, and although the innervation is not extensive in most dense connective tissues of the joint except for the capsule [80,97,98] and Hoffa’s fat pad [99], pregnancy-induced alterations to neuroregulation could also contribute to altered cell activity and tissue function.

Studies in a rabbit model have reported that there are alterations to vasoregulation during pregnancy mediated at the level of neuroregulation [100,101]. In addition, it has also been shown in a rabbit model that responsiveness to neuropeptides such as substance P (SP) and calcitonin gene-related peptide (CGRP) of the Achilles tendon (AT) [102] and the MCL [103] are altered during pregnancy. How this is manifested during pregnancy is not well-characterized, but it could be related to the functioning of cell receptors for neuropeptides on joint tissue cells and/or intracellular responsiveness to the signal-transduction systems in such cells. Of note, differences between males and females in responsiveness of AT tissue to neuropeptides was also noted [102], potentially indicating that sex hormones and/or puberty has shaped the responsiveness of the tissues in females to adapt to pregnancy. Thus, modification of neuroregulatory pathways could contribute to the altered functioning of joint tissues during pregnancy, in addition to potential direct effects of sex hormones on cells in the tissues. The influence of these variables such as neuropeptides and sex hormones are likely integrated into mechanotransduction responses as well, and all of these variables contributing to the success of establishing and maintaining the functioning of joints.

## 7. Aging and Senescence of Joint Tissues, Joint Functioning, and Joint Laxity

Joint laxity or looseness is known to decrease during aging, but it may be joint specific (discussed in [104]). With age, some joint tissues can get stiffer in both males and females, potentially due to increased cross-linking of collagen [105] and/or a loss of the boundary lubricant PRG4, which facilitates fibril sliding during loading of tissues such as tendons [42,43,44,45]. In contrast, PRG4 expression has been reported to increase in ligaments of the rabbit knee with age and following induction of surgical menopause [46,47]. As tissues of joints have receptors for sex hormones (discussed previously), and joint laxity in a subset of females is influenced by sex hormones [63,64], loss of hormones after menopause plus age-related changes in PRG4, as well as possible epigenetic changes due to use, participation in sports, or subclinical injuries preceding menopause and aging, could contribute to increased stiffness of joints and alterations to function.

## 8. Joint Laxity and Response to Injury

**Figure 2:**
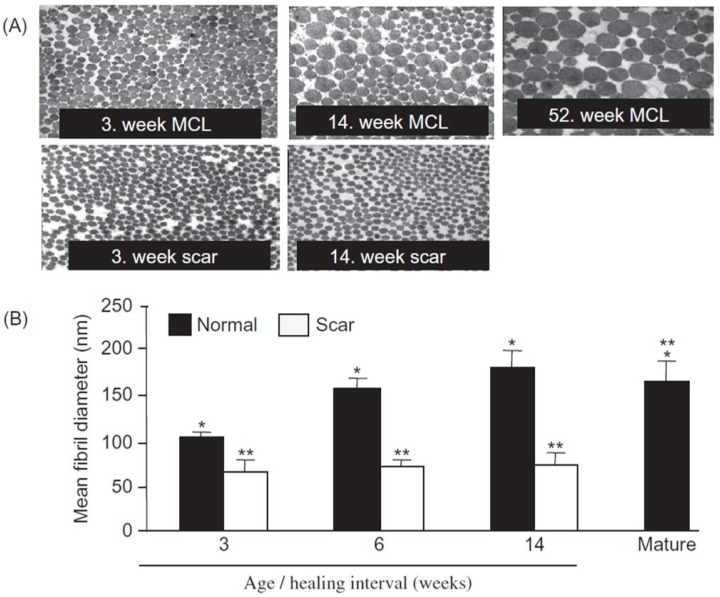
Failure of collagen fibril diameters to increase in the injured rabbit medial collateral ligament (MCL) during healing. (**Panel A**): Collagen fibril diameters in healing MCL scars at 3- and 14-weeks post-injury compared to those in the MCL during normal age-related maturation. (**Panel B**): Quantitation of collagen fibril diameters in the healing MCL compared to normal development. * *p* < 0.05 compared to corresponding normal development values; ** *p* < 0.05 compared to 52 week mature values. Reprinted with permission from Achari et al. [106].

Injury to joint tissues in otherwise healthy adult individuals leads to development of a healing response with formation of scar tissue in most tissues of a joint except for articular cartilage, which appears to have limited potential to heal, likely related to its avascular and aneural environment. In ligaments of the joint, the healing process leads to an altered composition and structure for a protracted period of time [5,106]. Tissues such as ligaments of the knee heal with increased levels of collagen III [5,106] and only small collagen fibrils for >1 year after injury and beyond (Figure 2). Thus, the healing response does not recapitulate the normal maturation process discussed previously. This leads to a tissue with increased laxity and low load behavior, and by extension a disruption in the integrity of the set point for the functioning of the joint as an organ system. In the case of a complete tear of the ACL, which cannot heal as the torn ends are no longer able to re-connect, reconstruction of the tissue using a graft of a tendon such as part of the patellar tendon or hamstring tendon is often undertaken. Initially, such grafts are mechanically stronger or as strong as the ACL, with large- and small-diameter collagen fibrils. However, over time, these autologous grafts become more scar-like with the appearance of small collagen fibrils [107], and they can develop creep and increased laxity [108,109,110], which results in an increased laxity of the tissue and the joint. Interestingly, it has been reported that there may be some differences between male and female recipients of such grafts for ACL repair [110]. This phenomenon of creep also occurs in preclinical models with an “idealized” ACL graft where the femoral end of the ACL is cored out and then re-inserted into its initial site and anchored [111]. While idealized mechanically, the process of coring out the femoral end of the ACL appears to lead to disruption of the vascularity and innervation of the tissue as well as joint inflammation [112], factors which likely contribute to its ultimate compromise.

Therefore, injury to joint tissues leads to a healing process that does not recapitulate normal maturation. Scar tissue that forms after injury is compromised both biologically at the level of collagen fibril diameters and mechanically, leading to increased tissue and joint laxity. PCR analysis of scar tissue has revealed that the cells in such tissues are expressing some relevant molecules inappropriately compared to normal tissues [106]. Attempts to improve on normal healing processes using anti-sense gene therapy approaches employing antisense to decorin, which binds to collagen I, led to some large collagen fibrils in rabbit MCL scar tissue and partial improvement in function, but the response was not consistent [52,53], likely due to an inability of the delivery method to affect all cells in a somewhat dense connective tissue [56]. This area of improving outcomes while knowing some of the molecular and mechanical deficiencies of scar tissue is deserving of additional investigation and research, and the outcomes may have application to improving joint hypermobility as well.

The injury site for joint tissues such as ligaments is also dependent somewhat on the age of the individual, with avulsion injuries more common in children [113,114] and skeletally immature animals such as the rabbit [115,116]. In contrast, injuries to ligaments in skeletally mature individuals usually occur in the mid-substance of the tissue [115]. Thus, before skeletal maturity, the transition point at the entheses of joint tissues are likely the weakest points in the growing tissues. However, avulsions can also occur in adults.

**Figure 3:**
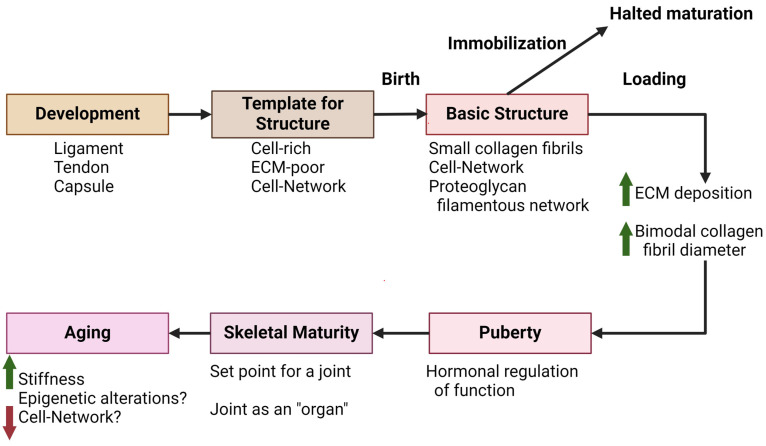
Outline of normal joint tissue development, growth, and maturation across the life span. At each step in the process, some of the relevant consequences are also indicated, with some still in need of further investigation indicated with a question mark.

In summary, development, growth, and maturation of connective tissues of a joint occurs in a coordinated manner, leading to an integrated organ system (Figure 3). The successful formation of a mature joint with an appropriate structure and biomechanical properties is then posed to accommodate stressors placed on them over the course of the ensuing decades of life.

## 9. Characteristics of Joint Hypermobility Syndromes

### 9.1. Mutations and Phenotypes

The joint hypermobility syndromes include a number of syndromes that are defined by genetic mutations, such as most of the Ehlers–Danlos syndromes, Marfan’s syndrome, Loey–Dietz syndrome, and others, as well as those functionally defined, but mutations in specific genes have not yet been identified (i.e., hEDS). The mutations involve a diverse set of molecules and include the fibrillins in Marfan’s syndrome, as well as mutations in several collagens, enzymes, TGF-beta and receptors, proteoglycans, and tenascin-X (reviewed in [6,9,117,118]). The frequency of cases of joint hypermobility is estimated to be 1/ < 1000, so the condition is very common for as yet unknown reasons. How this breadth of known mutations contributes to joint hypermobility as a common phenotype remains to be elucidated and will be discussed below.

Those with most of the mutations characterized exhibit joint hypermobility but also can have skin hypermobility and higher risk for cardiovascular events such as aneurysms and aortic dissection that can be fatal (reviewed in [6]). However, in those with hEDS, the primary feature is joint hypermobility. Many of those with these syndromes have abnormal collagen fibrils in their connective tissues (reviewed in [6]), but the data has been primarily derived from skin biopsies and may not reflect the status of collagen assembly in other tissues such as ligaments, tendons, and capsules of joints. As shown in Figure 1, the maturation of collagen fibril diameters in such tissues appears to be tissue-specific, possibly in relation to the loading requirements of the tissues as the individual grows and matures.

Individuals with JH often experience joint pain [16,17,18,119] and also have a high incidence of fibromyalgia [120,121,122]. These individuals are also at risk for joint injuries and fractures [123,124].

### 9.2. Challenges and Limitations in Knowledge

It is challenging to diagnose JH in most young individuals as during rapid growth before puberty many individuals in this age bracket have fairly lax joints [58,59]. However, after puberty and into adolescence and adulthood when those without a JH diagnosis establish a setpoint for the function of their joints, the laxity becomes stabilized. Thus, a JH syndrome can be better diagnosed when a stage in the lifecycle is reached where it is more readily distinguished from those without a clinical JH. Whether females with a JH diagnosis also experience menstrual cycle variation in joint laxity remains to be defined and should be the topic of future investigation.

As it is ethically challenging to assess tissues from individuals with an identified JH syndrome other than perhaps skin biopsies and blood/serum samples, research into the impact of defined mutations on specific joint tissues is limited and relies in part on non-invasive imaging modalities, and possibility the use of preclinical models containing specific mutations analogous to those occurring in humans. Of course, a limitation of the latter is that the model is imposed on an animal background, usually a rodent such as a mouse or rat. In the case of an inbred mouse, the background genetic makeup of a mouse strain may affect the phenotype of any specific mutation. However, humans are very heterogeneous, so investigations using mouse models should optimally use several strains to capture the influence of background genes as mouse strain selection can influence outcomes [125,126].

Based on the above literature, joint hypermobility can result from a number of mutations in genes relevant to joint tissue ECM, but some forms (i.e., hEDS) remain undefined at the molecular level. How such a diverse array of affected genes can result in a common outcome (joint hypermobility) through all of the stages of life remains to be elucidated. Whether there is a single pathway to this outcome or multiple pathways to achieve the same result also remains to be determined.

**Figure 4:**
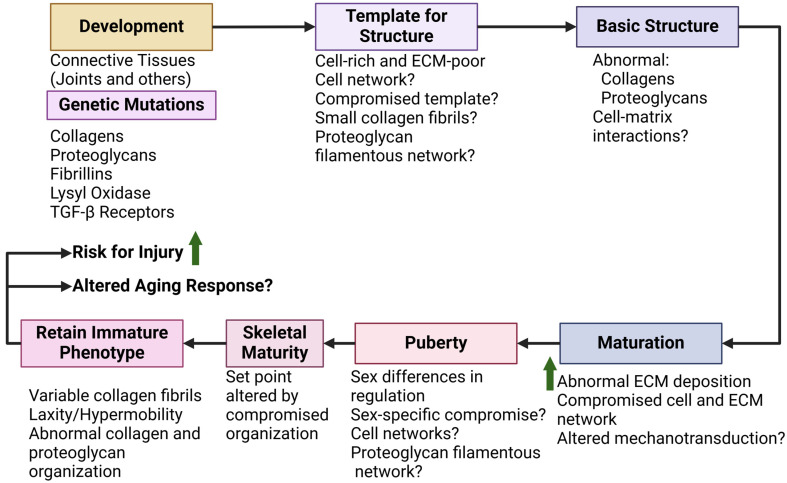
Summary of salient known and hypothesized features of joint tissue development and maturation in individuals with Joint Hypermobility syndrome. The consequences of the mutations associated with the various steps in growth and maturation possibly contributing to the compromised function are also indicated, and those in need of further research are indicated with a question mark. The outline presented features common characteristics rather than details of each known subtype of the condition.

The following discussion focuses on what is known and what could be the focus of future studies that would also impact our understanding of regulation of function in those without a JH syndrome. A generalized outline of steps in the development, growth, and maturation of joints and joint tissues in JH syndrome is depicted in Figure 4 to assist the reader and allow comparisons to analogous steps in those without JH depicted in Figure 3.

## 10. Possible Lessons Learned and to Be Learned from Individuals and Relevant Preclinical Models of Joint Hypermobility Regarding Joint Function Across the Life Span

While it is important to understand the basis for the functional alterations or phenotypes associated with a JH syndrome in order to devise interventions to improve the quality of life for such individuals and reduce their risk for injury and perhaps degenerative joint diseases of aging, the study of such individuals also offers insights into how “normal” processes regarding joint function are regulated and governed during different stages of the life cycle. How joints and joint tissues arise, mature, and progress through the life span are not well-characterized in the face of disruption of a number of relevant molecules either as identified mutations or associated with dysfunction of molecular regulation [6,9,127,128], often leading to pain (reviewed in [129]). Thus, for EDS, hypermobility type, no specific mutations have been reported, but perhaps the regulation of some important molecules such as specific collagens are compromised [127] and may require unique interventions for management [130]. This could occur at the level of the promoter region of the genes in question, or in the responsiveness to normal growth regulators at the level of their membrane receptors or signal transduction as possibilities. However, this issue will require further research to elucidate the mechanisms involved.

### 10.1. Development

Despite the myriad number and type of known molecular alterations that can contribute to joint hypermobility [6], the joints of individuals ultimately affected by such conditions still appear to cavitate and develop with a fairly normal template of ECM and cell populations, and except for a minority of individuals severely affected as children, nearly all individual with a JH syndrome have functioning joints that operate to allow for mobility and navigation through the environment, but with some limitations. In addition, in some tissues such as articular cartilage, some collagen variants are used during development and then replaced as the tissue grows and matures after birth. While development in affected individuals appears to be fairly normal based on the outcomes after birth, whether there are subtle alterations to both the ECM and their cell networks (discussed in [128]), including the innervation of the various joint tissues, remains to be determined. Of particular interest is the finding that there can be collagen fibril diameters of large but with somewhat irregular surfaces and organization can still have compromised low and high load properties (discussed in [128]).

Alternatively, and potentially dependent on the repertoire of background genes, the use of fetal-specific variants of splice-variants of affected or non-affected genes could lead to fetal organization of the ECM that appears normal [131,132,133,134] that then becomes different after birth both in composition and organization [132,133]. Thus, the development and pre-birth composition and organization of connective tissues of the joint could be relatively normal and only compromised after birth and during growth and maturation, perhaps involving an inability to switch from the fetal molecular forms to the more mature forms. In the case of Marfan syndrome, a large number of mutations have been documented along the length of the fibrillin gene product, and some genotype-phenotype relationships have been proposed (reviewed in [135]). However, most of the genotype-phenotype relationships that have been identified are for only non-joint hypermobility manifestations as far as could be determined from the literature.

A second possibility is that via redundancy in ability to perform specific functions in providing a useful ECM template, some molecules could initially substitute for a defective molecule to form a structural template but not be able to maintain the ECM requirements occurring during the rapid phase of growth happening after birth. An example of a set of molecules with redundancy is the SLRPs (i.e., decorin, biglycan, lumican, fibromodulin). Elements of this family can interchange functionally under some conditions and affect collagen fibrillogenesis and connective tissue functioning [136,137,138,139,140]. These molecules can bind to relevant collagens, enzymes (i.e., lysyl oxidase), growth factors (i.e., TGF-beta), and one member of the family, decorin, has been implicated previously in joint hypermobility syndromes (discussed in [7]) but has not received much attention recently. However, care in such investigations should be taken regarding interpretations as in fibromodulin knockout mice, there is a fourfold increase in lumican expression and deposition in tendons [141], a compensatory response that could cloud the interpretation of results.

Exploration of such variables in the development of the various forms of genetic and functional disorders of joint mobility could be addressed using rodent models with the caveat that some aspects may be strain-specific and dependent on background genes and their regulation [125,126]. In addition, there may be some species-specific aspects to the phenotypes identified involving molecular redundancy, splice-variant generation, and molecular mimicry that could explain some of the tissue involvement, extent of the hypermobility, and other features involved in the heterogeneity of presentation in individuals affected. While some of the heterogeneity in the phenotypes associated with syndromes leading to hypermobility and other manifestations have been identified [135], others could possibly be due to background gene usage and dorsal-ventral patterning or other explanations.

### 10.2. Growth and Maturation

In the face of a spectrum of known mutations, as well as phenotypes without known mutations (i.e., hEDS), the joints of individuals with hypermobility phenotypes grow and are able to function within limits. This implies that many aspects of growth and maturation are retained in the presence of mutations in important molecules contributing to ECM composition and organization. This is of particular interest in the case of Marfan’s syndrome with mutations in the fibrillin gene. Such individuals are often of tall stature (discussed in [142,143]), particularly in females. Thus, even with extensive bone growth, the soft connective tissues of the joints can respond, leading to functional joints albeit with altered laxity. While laxity is likely mainly a low-load property of these tissues focused on proteoglycan content and organization [95], this implies that in many joint soft connective tissues, the content and organization remain sufficient to maintain some aspects of joint function. However, individuals with some hypermobility syndromes are also at higher risk to develop overt joint injuries and thus may also have compromise in aspects of high-load properties (i.e., in collagen composition and organization-fibril diameters) as well as low-load characteristics (discussed in [128]). Elucidation of details regarding the association of specific mechanical properties should be the focus of future research efforts.

From the above discussion, such research could focus on the cell networks in the soft tissues and innervation patterns as they are critical for joint functioning [23,24,25]. Certainly, the interaction of connective tissue cells with matrix components via cellular integrins is known to influence how cells respond to signals and express ECM molecules in response to loading (reviewed in [144,145,146]). As the mutated proteins must be expressed to exert their influence, directly and indirectly, cell signalling via cells and cell networks may be critical elements in the regulation of the tissues. Similarly, as soft connective tissues of joints are innervated [147] and endogenous cells can respond to neuropeptides [101,102,148,149,150], this regulatory system could also be potentially influenced by mutated proteins in the ECM.

Finally, the integrity of the muscle system in children with heritable connective tissue syndromes with joint hypermobility has been reported to be compromised in these children [151], as well as in bone [152]. Such children have been found to have a lack of physical fitness and decreased muscle function. This may be due to intrinsic defects in muscle development or due to inactivity associated with development of pain on exercise or the fibromyalgia mentioned above. In Marfan syndrome, this could be the result of dysregulated TGF-beta signaling associated with mutations in the fibrillin gene [152]. Whether such dysregulation is associated with specific mutation clusters in the fibrillin gene could not be found in the literature. However, given the importance of TGF-beta as an anabolic modular of growth in many tissues, this could be the focus of future studies. Whatever the combination of factors is that contribute to the compromised muscle integrity, it appears to be carried forward into adulthood, particularly in females [153,154].

Therefore, several aspects of joint development and function during the phase of the life cycle associated with fairly rapid growth and maturation progress normally in individuals with joint hypermobility associated with genetic mutations, as well as those with hEDS. Thus, the spectrum of mutations in molecules that are very relevant to effective ECM composition and structural organization (i.e., collagens, proteoglycans, growth factors, fibrillins, tenascins, enzymes) still leads to a functional joint, albeit joints with an altered phenotype. Details regarding how this can be accomplished in the presence of different mutations could lead to not only new approaches to intervene early with such individuals, but also to a better understanding of how normal development followed by growth and maturation is regulated.

### 10.3. Puberty

With the onset of puberty, there is the onset of both menstrual cycles and another round of growth that is in part, sex dependent. Some features of growth were reported to be different from those without a genetic disorder in individuals with Marfan syndrome [155], and a subset of individuals with EDS were reported to have an altered menstrual bleeding pattern [156]. However, no studies examining alterations to joint laxity or hypermobility during the menstrual cycle in such individuals could be found in the literature. This lack of information may be due in part, to the reported higher incidence of dysregulated menstrual cycles in individuals with hEDS [157], which would potentially complicate obtaining definitive data.

Of potential relevance to this issue are reports that female athletes exhibit less knee laxity than non-athletes [69], likely due to enhanced muscle control of the joints, while those with hypermobility are usually less fit [151]. Being less fit with less muscle regulation of laxity may compound the situation across the menstrual cycle and assessment of increases in laxity associated with the menstrual cycle. Being an athlete or not did not affect progesterone or relaxin levels across the menstrual cycle, and these molecules did vary with the highest levels observed in the luteal phase [69]. Of note, serum relaxin levels have been reported to be higher in females with what was called benign hypermobility syndrome than in those without [158], but the menstrual cycle was not mentioned. In addition, if verified that individuals with specific subtypes of joint hypermobility have elevated serum levels of relaxin, longitudinal studies should be commissioned to address when in the life span (i.e., growth and maturation, puberty, or skeletal maturity) such increases occur, and if there are sex differences in this development. However, as relaxin has been implicated in collagen metabolism (discussed in [158]), and collagens such as collagen I is more associated with high load behavior of joint tissues while proteoglycans low load behavior [95], such elevations in relaxin may help explain the risk for joint injuries in several subtypes of those with JH. A combination of low and high load compromise that is complementary could increase the risk of injury and pain for such individuals. As the mechanisms responsible for systemic increases in relaxin (i.e., serum levels) are not known, this aspect of the role of relaxin in joint hypermobility should be re-visited using targeted DNA sequencing approaches.

Further attention to whether there are increases in joint laxity/hypermobility during the menstrual cycle is likely warranted in individuals with JH. Furthermore, it would be important to know if menstrual cycle-dependent changes may be additive to that observed in individuals with JH, or if there is an “upper limit” to laxity beyond which it would lead to overt joint damage and elevated injury risk.

### 10.4. Skeletal Maturity

At skeletal maturity, growth plates close and the integrated function of tissues of a joint reach a set point. This set point for individuals with JH means that for the next several decades, the hypermobility is somewhat fixed, except for the muscle component, which is likely adaptable by exercise, at least in those without JH [69]. This latter point is likely relevant as muscles appear to be affected in adult females with some forms of EDS [159,160]. After skeletal maturity, the two most prominent aspects of risks for adverse events in individuals with various forms of JH is joint injury and for females, pregnancy.

Injury to joint tissues can take many forms, depending on the extent of the hypermobility, which joints are affected, and whether the conditions are symptomatic or non-symptomatic (i.e., pain), as those with pain may be more risk adverse than those without pain. Injury to joint tissues can be acute (i.e., dislocations, subluxations, tissue tears) or chronic (i.e., tendinitis/tendinosis, overuse injuries) [161,162], where joint instability and overuse may also lead to degenerative conditions such as osteoarthritis later in life. As wound healing is reported to be compromised in individuals with various forms of EDS, LDS, and Marfan’s syndrome [163,164,165,166,167], one would expect that injuries to essential joint tissues would heal less effectively than in those without such conditions, and thus, the joint hypermobility may become more evident after an injury to ligaments, joint capsules, or tendons and could be assessed using available tools. Also, whether such joint injuries occur non-randomly across the menstrual cycle was not evident from the literature examined. These points should be addressed in future investigations.

Pregnancy in those with various forms of EDS, LDS, and Marfan syndrome poses many severe threats to the mother, and joint hypermobility is not of primary importance compared to aortic dissection risk, for example [168,169]. However, a large number of reports have focused on the impact of pregnancy on those with the hypermobility type of EDS or hypermobility spectrum disorders [170,171,172,173,174]. These reports indicate a high level of successful pregnancies and no obvious increases in joint injuries that may have been expected if significant increases in joint laxity or mobility had occurred. In some reports, there were indications that caesarian sections were used to avoid the potential for dislocations associated with a natural birth, but whether that decision was based on pregnancy-associated increased risk was not specifically mentioned. Therefore, it is not clear from the literature whether there are pregnancy-associated increases in joint laxity or mobility in this population, or the excessive laxity already present obscured its detection. This should be further investigated by future studies.

There is also a paucity of information available regarding lactation and milk quality in maternal populations with various forms of EDS [175]. While there could be connective tissue challenges regarding lactation in such populations, another issue relates to the milk itself. That is, is there relaxin in the milk of such patients if they have excess relaxin in serum when non-pregnant [158]. It will be important to assess relaxin levels in the serum of such individuals while non-pregnant and pregnant and then assess relaxin levels in the milk. In canine systems, levels of relaxin in the milk of some mothers are high and can be transmitted to the pups via milk and increase risk for excessive joint laxity, particularly of the hip [176,177]. While no information could be found in the literature regarding levels of hip dysplasia in the offspring of hEDS mothers, one might have expected some mention if it occurred with high incidence. This area could be the focus of future investigations.

### 10.5. Aging and Senescence of Joint Tissues and Joint Function in Joint Hypermobility Syndromes

As discussed in Singh et al. [59] and Aronson et al. [58], most joints of individuals without the various forms of genetic and non-genetic hypermobility syndromes become stiffer with age. This is likely due to collagen cross-links and other changes to the matrix of tissues such as tendons, ligaments, and others. The question then arises as to whether similar changes to joints occurs in people with such syndromes as they have compromised ECM function. When speaking to patients with such conditions, they remark that their joints appear to become stiffer after ~40 years of age [Hart, unpublished observations], but a search of the literature has not revealed any studies documenting such changes.

### 10.6. Response to Injury

Individuals with various forms of EDS, LDS, and Marfan’s syndrome often heal poorly and with altered scarring and healing processes (discussed in [128,163,164,165,178,179]). Most of such observations relate to the healing of skin injuries. Similarly, wound healing of various tissues in mouse models of EDS and related conditions are also reported to be compromised [128,166,180,181,182]. Most reports discuss or assess the cellular processes of healing (i.e., fibroblasts, inflammatory cells, macrophages) and the outcome of healing (i.e., delays, collagen fibril alignment, altered histology, biomechanics). However, evaluation of collagen fibril diameters following injury and healing were not found. This would be an important aspect to focus on in future studies as wound healing in rabbit models without connective tissue diseases leads to compromised healing with small collagen fibrils for an extended period of time (Figure 2). At the molecular level, healing of the rabbit MCL does not recapitulate normal development and maturation [106], and scars compromised biomechanically for an extended period of time [5,96] but the collagen fibrils do re-align with the direction of the loading [183].

While injuries in both adults and mouse models of connective tissue diseases heal poorly, they do heal, particularly incisional skin wounds, so if they start with a compromised tissue, the scar tissue may be further compromised. However, there are reports of ACL reconstructions in EDS patients using autologous graft tissue that resulted in good functional repair [184,185,186], and with maintenance of joint stability for several years. Therefore, starting with what is potentially a compromised tendon tissue and using it to functionally reconstruct the ACL led to healing of the insertion sites and a return to relative joint stability. The authors did not mention if the reconstructed knee ultimately developed increased laxity that could have been due to the development of creep in the graft. Thus, there may be location-specific aspects to healing outcomes in EDS patients.

As noted previously, ligament ruptures in patients without hypermobility syndromes often occur as avulsions in the skeletally immature and in the mid-substance of the skeletally mature. In those with hypermobility disorders, avulsions are again common in the skeletally immature, and reconstructions often require revisions (discussed in [187]). Whether skeletally mature individuals rupture ligaments mainly in the mid-substance of the tissues could not be found in the literature, but this may be assumed since it has not been noted otherwise.

Further characterization of wound healing in different locations, and in the presence of different mutations in both humans and experimental models, could lead to new insights into both how and why they differ from healing in those without such mutations or hypermobility conditions. Such information may be very useful in the design of interventions to overcome processes compromised by mutations, aging, or comorbidities such as diabetes.

## 11. Summary, Conclusions and Speculations

From the above discussions regarding the regulation of joint function in those without hypermobility and those with a clinical diagnosis of hypermobility, there are a number of points that can be made. These include the following:

(1). Joints can develop, grow, and mature in the presence of many different mutations in critical molecules. The basic templates for the various joint tissues must be able to form and develop their coordinated activities to allow the joints to exhibit the basic functions of a particular joint in the presence of such mutations. While those with hypermobility have joints that are compromised in some respects, they still function in a fairly well-controlled manner. This implies that neurocontrol and neuroregulatory systems are functionally fairly intact. As well, in those that have clinical hypermobility with an identified or as yet unidentified molecular basis, they are still able to respond to the anabolic environment of growth, albeit with an altered outcome. Thus, there is considerable flexibility in the development of joints and joint tissues as evidenced by the breadth of the known genetic mutations that give rise to clinical phenotypes. However, the critical element here is the detection of a clinical phenotype, and the mutations identified may only be the “tip of the iceberg” regarding the molecular basis for the normal range of variation in joint laxity and mobility where the impact of a mutated molecule or regulatory process does not lead to a clinical diagnosis.

(2). The impact of known genetic mutations is heterogeneous even within families with regard to which tissues are affected (i.e., joints and/or tissues of the cardiovascular system, with the latter posing a more significant risk for lethal complications). Such situations may indicate that the genetic complement of background genes allow for redundancy and/or use of non-mutated molecules to compensate for a mutated protein in some tissues. Other variations could result from expressed mutated proteins with partial function, use of splice variants that eliminated mutations in some exons during development, variations arising from ventral-dorsal patterning during development, or even sex-dependent use of specific molecules. Thus, there are several potential opportunities to contribute to flexibility and options in the development of joint tissues. Such flexibility could lead to variations in joint functions and responses to specific stimuli without leading to a clinical phenotype.

(3). In young individuals/children, many joints exhibit more laxity than as adults and it can be difficult to distinguish those having joint hypermobility disorders from those without such conditions. However, as they age, in those without such a condition, the joints mature with regard to laxity until skeletal maturity with establishment of a set point that provides the joint with stability. In contrast, in those with hypermobility syndromes, the joints continue to be very lax, and they appear to not mature, leading to joint instability and pain. Given the breadth of mutations in a variety of molecules important to ECM structure and function, this commonality of an apparent failure to mature but still maintain sufficient structure to function, although compromised, as an organ system is quite remarkable. As low-load behavior in normal joint tissues is related to proteoglycan composition and organization and high-load behavior related to collagen composition and structure [95], some individuals with joint hypermobility disorders not only have excessive laxity but also increased risk for injury. In such individuals, there may be a combination of compromised low- and high-load behavior in relevant joint tissues such as ligaments and perhaps joint capsules. Thus, in the joint hypermobility population the impact of the mutations of dysregulated processes is different from what is regulating normal joint tissues. However, injuries to normal joint tissues such as ligaments leads to a scar tissue that is functionally compromised for an extended period of time due to wound healing not recapitulating normal development. While injury to normal tissues such as the rabbit MCL leads to collagen fibrils of small diameters for an extended period of time (Figure 2), collagen fibrils in tissues from those with joint hypermobility can be quite variable in fibril diameters depending on the mutations involved [6], indicating that hypermobility is not consistently correlated with small collagen fibrils. However, in JH patient populations, the collagen fibril diameters are usually determined in skin samples, so this issue needs to be pursued in more detail with additional research.

In addition, in both those with and without a joint hypermobility disorder, muscles play a role in joint laxity, but in many individuals with a joint hypermobility disorder, the muscles are often compromised as well, and they can also have fibromyalgia [120,121,122]. For those individuals, it may be possible to “correct” some of the joint hypermobility through effective neuromuscular training programs [16,17,130].

At the present time, there are no interventions to correct the compromised soft tissues of joints such as the knee for those with joint hypermobility, and attempts to use antisense gene therapy to improve ligament healing in rabbits was met with many challenges. Therefore, it would appear that the most effective interventions to improve joint stability in those with joint hypermobility disorders is to focus on what is likely the most adaptable tissue, the muscles and muscle function via targeted exercise programs, building on current approaches (reviewed in [188,189]).

(4). Injury to joints already compromised by mutations to ECM molecules or dysregulated joint mobility (i.e., hEDS) heal poorly compared to injuries to joint tissues without hypermobility (i.e., “normal” healing). However, normal healing can be quite heterogeneous in outcome (discussed in [190]), and is influenced by age (discussed in [191,192]) and genetics (discussed in [193,194,195]). Therefore, it is still to be determined whether compromised healing in those with various forms of hypermobility syndromes is due to a direct effect of the mutations or is an indirect effect via the consequences of the conditions. Further to this point is the fact that wound healing outcome may depend on the location of the injury and the tissue injured [196], and nearly all wound healing in hypermobility syndromes has been focused on skin wound healing. While location-specific healing outcome has been shown in pig models (i.e., skin vs oral cavity) [196], influences due to genetic differences in similar models have been consistent when skin vs ligament healing was compared. Therefore, issues regarding healing outcomes in joint hypermobility syndromes vs normal joint tissue healing remain to be resolved. Future studies should explore variables including sex, as estrogen and estrogen receptors have been implicated in healing outcomes [197,198].

(5). Joint laxity can vary in many females without hypermobility syndromes during the menstrual cycle or during pregnancy, and this is associated with increased risk for joint injury. Females with some hypermobility syndromes appear to be at risk for connective tissue complications during pregnancy, but issues regarding joint laxity during pregnancy and the across the menstrual cycle are mainly anecdotal and it is not clear as to the extent and whether it is associated more or less with specific forms of joint hypermobility. For many of the joint hypermobility syndromes, the impact on quality of life and extent of disability is greater in females than males. This could be related to sex hormones and regulation of joint tissues themselves or other regulatory systems such as inflammation that exhibits sex differences (reviewed in [70]). However, further research to determine and quantify variation in joint laxity across the menstrual cycle and during pregnancy should be performed in these populations as the findings could provide information as to the limits of variation in joint laxity that can occur. This may be relevant to whether exercise programs in these populations should be restricted to certain times in the menstrual cycle.

(6). The question of why joint hypermobility syndromes are so prevalent in populations around the world remains to be answered. While not all joint hypermobility syndromes have been identified as Ehlers–Danlos subtypes, Marfan syndromes or Loey–Dietz syndromes, and many are not associated with a defined genetic mutation, the prevalence of joint hypermobility as defined by scores on the Beighton scale has been reported to be 2–57% [199], depending on the population being assessed [59,199,200]. Some individuals with Marfan syndrome, vEDS, or other syndromes have a high incidence of severe cardiovascular events, while many with joint hypermobility are not severely compromised. However, some with hEDS can be very compromised by pain and joint injuries. As most studies would indicate females are affected by joint hypermobility more so than males, one may try to interpret sex differences to an association of joint hypermobility with reproductive success such as childbirth. However, given the ethnic variation reported for joint hypermobility [59,199,200], it is not clear why such significant ethnic differences would persist if hypermobility was advantageous for reproduction throughout evolution. Also, given the breadth of the known mutations in molecule type (i.e., collagens, proteoglycans, tenascins, fibrillin) and their function in joint tissues contributing to joint hypermobility, it remains a conundrum of how they all focus on joint hypermobility as a common outcome. Thus, the answer may lie in the consequences of the known mutations rather than the actual mutations themselves. Relevant to this point is the finding that the gene for biglycan, one of the SLRPs known to have a large number of biological functions in different tissues, including regulation of collagen fibril assembly [54,201,202,203,204,205] is located on the X chromosome and subjected to X inactivation in females [206]. Furthermore, it can be glycosylated differently in different environments [207]. Thus, additional research focus should be on SLRP metabolism in joint hypermobility syndromes, including that related to biglycan, decorin, lumican, and fibromodulin in the search for common mechanisms in joint hypermobility.

(7). From the discussion in this review/perspective, in individuals with hypermobility syndromes joints arise during development fairly normally. They then progress through post-birth growth and maturation leading to joints that can function as an organ system, but with the caveat that they remain very lax/hypermobile leading to a skeletally mature state with the joints remining hypermobile and often with increased risk for overt injury. One interpretation of this scenario is that the joints of affected individuals have many features in common with joint development and maturation in individuals without joint hypermobility but fail to mature properly. Normal maturation requires integration of each component of the joint as an organ system as they grow and respond to biological and biomechanical signals. As such, the ECM of each tissue is a reflection of how the cells in the tissues respond to biological signals such as anabolic growth factors and hormones, as well as biomechanical signals transmitted to cell outputs via mechanotransduction (reviewed in [208]). Clls in the tissues are associated with matrix components via integrins and membrane lectins and respond to load-induced deformation of the matrix with sensing molecules such as piezo-1 and -2 [209,210,211] or transient receptor potential channels such as TRPV4 [212,213].

Thus, for normal growth and maturation, loss of appropriate sensing or compromise of such systems to sense biomechanical signals will lead to a loss of growth even if biological signals are present. This was shown by immobilizing the knees of rabbits while they were still very young [21,31]. Immobilization and loss of biomechanical stimulation led to complete arrest of growth, so tissues of the knee appear to require biomechanical stimulation in order to respond to systemic anabolic growth factors, and immobilization may have also led to loss of growth factor receptor expression or function. Of note, re-mobilization of the knee after a period of immobilization led to a partial recovery of size and function [31], so re-initiation of the biomechanical environment led to a return of some aspects of growth and maturation. Thus, biomechanical stimulation of tissues of the joint appears to override biological signals during critical stages of growth and maturation.

The implication of this scenario is that cells in joint tissues of individuals affected by syndromes such as EDS, Marfan’s, LDS, as well as hEDS, are not responding to biomechanical signals appropriately, but some joint features related to growth are still occurring. The possibility that the influence of mutations and alterations in other syndromes such as hEDS leads to compromised cell-ECM regulation of mechanotransduction has been raised by Malek & Koster [214]. In normal tissues, the cells are interacting with molecules in the ECM via members of the integrin family. The transmembrane integrins are also linked to the cytoskeleton of the cells. When a tissue is loaded by tensile, compression, or shear forces, this leads to deformation of the tissue and the cells linked to the matrix. The cytoskeleton resists deformation but can adapt to loading as per the tensegrity model (discussed in [215,216]). Deformation of cells in response to loading (discussed in [217,218]) leads to changes in gene expression and thus, the integrity of such a system is central to growth and maturation of these tissues. Similarly, loss of such mechanical stimulation is not a null event, as the cells in a tissue respond to such conditions by derepressing expression of a catabolic cascade of genes leading to tissue atrophy [219]. Therefore, disruption of the integrity of the cell–ECM interaction and compromise of mechanotransduction systems could lead to alteration in the anabolic/catabolic balance during the critical time of maximal growth and maturation. If the interpretation presented by Malek and Kostner [214] regarding the functional alterations in JH are based on disruption of mechanotransduction systems is correct, then how mechanical signals are affecting some tissue properties and not others will be interesting to resolve with future research. Such research may again involve the study of SLRP metabolism, as such molecules not only interact with other matrix molecules but also cells [220,221,222,223].

While the focus of this review/perspective piece has been on joint hypermobility, this concept of a failure of tissues to mature in the presence of a diverse set of mutations due to compromise in cell responses to biomechanical signals could also be applied to those affecting the integrity of cardiovascular tissues as well [212]. Furthermore, mutations in Marfan syndromes of fibrillin may also affect mechanotransduction [224,225], so tissue-specific loss of ability to respond to mechano-signaling may be a common feature of several different syndromes. How such tissue-specific variation arises and the mechanisms underlying such variations could be very informative for understanding of tissue regulation in a broader sense. Further research on this concept of disruption of mechanotransduction playing a central role in the phenotypes associated with these syndromes could lead to insights into regulation in normal tissues as well.

While continued comparison of joint function and response to stressors such as age and injury, as well as a changing hormonal landscape in those with and without joint hypermobility syndromes, will provide new information regarding the range of regulatory detail that define joints as organ systems, there are areas where additional studies will potentially fill in some of the existing gaps in the knowledge base and possibly help in the development of interventions to assist those with hypermobility syndromes as well as those without clinical syndromes. Thus, comparing current and future knowledge bases between different populations (i.e., those with and without hypermobility) may provide key information regarding the regulation of joints and joint tissues that could lead to improved understanding and enhance the development of effective interventions.
